# Grazing on Upland Pastures Part-Time Instead of Full-Time Affects the Feeding Behavior of Dairy Cows and Has Consequences on Milk Fatty Acid Profiles

**DOI:** 10.3390/ani9110908

**Published:** 2019-11-01

**Authors:** Elisa Manzocchi, Madeline Koczura, Mauro Coppa, Germano Turille, Michael Kreuzer, Joel Berard

**Affiliations:** 1 Institute of Agricultural Science, ETH Zurich, Universitaetstrasse 2, 8092 Zürich, Switzerland; elisa.manzocchi@usys.ethz.ch (E.M.); michael.kreuzer@usys.ethz.ch (M.K.); 2 Université Clermont Auvergne, INRA, VetAgro Sup, UMR 1213 Herbivores, F-63122 Saint-Genès-Champanelle, France; madeline.koczura@inra.fr (M.K.); mauro.coppa@inra.fr (M.C.); 3 Institut Agricole Régional, 11100 Aosta, Italy; zootecnia@iaraosta.it; 4 ETH Zurich, AgroVet-Strickhof, Eschikon 27, 8315 Lindau, Switzerland

**Keywords:** mountain pasture, grazing behavior, fatty acid, milk coagulation, milk quality, dairy cattle

## Abstract

**Simple Summary:**

Transhumance of dairy cows to upland pastures during summer is a tradition in mountain farming systems. Different management systems are practiced in the upland depending on topography and available infrastructures. This study aimed to assess the effects of two traditionally practiced upland pasture management systems (full-time grazing and part-time grazing) on feeding behavior and milk yield and quality. Cows with access to pasture for only 6 h per day had different feeding behaviors than cows on pasture 24 h per day; their milk yields, however, were similar. Although protein and casein contents were higher in the milk of cows with full-time access to pasture, milk coagulation properties did not differ between the two systems. The differences found in milk fatty acid profiles suggest that cows with part-time access to pasture mobilized more body fat reserves to counterbalance the energy expenditures required during fasting periods and for walking back and forth between the barn and the pastures.

**Abstract:**

Different grazing management systems are practiced on upland dairy farms during summer, depending on topography, local traditions, and infrastructure. The present experiment compared two distinct management systems with respect to feeding behavior and milk-related properties. Two similar groups of eight Valdostana Red Pied cows originating from two farms were followed during three grazing events in summer on three upland grazing sites. Cows in the full-time grazing group were kept exclusively on pasture and milked twice daily in a mobile milking parlor. Cows in the part-time grazing group had access to pasture for 4 h and 2 h after their morning and evening milkings, respectively. The part-time grazing cows differed markedly in their feeding behavior; they exhibited shorter daily ingestion times and longer durations of ingestion and idling bouts than full-time grazing cows. Part-time grazing cows had lower milk protein and casein contents, but milk yield and milk coagulation properties did not differ from the full-time grazing cows. As a result of the fasting periods in the barn, part-time grazing cows synthesized less fatty acids de novo and mobilized body fat reserves, as evidenced by the higher proportion of oleic acid in their milk fat.

## 1. Introduction

Mountain farming systems often include a summer grazing season with so-called vertical transhumance, which helps valorize upland pastures. Such systems are typically associated with the on-farm production of high-value cheeses, which are often labeled as protected designation of origin (PDO). Different management system alternatives are used to exploit the upland pastures and are adapted according to herd size, topography, and the availability of infrastructure elements (e.g., roads, buildings, electric power grid); they are also frequently related to local traditions. In the Alps and the French Massif Central, one widespread management system is full-time grazing, where cows are outdoors day and night and are milked on pasture in mobile milking parlors [1,2]. Alternatively, part-time grazing houses the cows in a barn equipped with milking devices that is close to a cheese dairy. This part-time grazing system is traditionally used to avoid transporting milk over long distances, often due to a lack of roads and difficult topography. According to farmers, the part-time grazing system also offers some shelter to the cows against unfavorable climatic conditions [3]. However, housing the cows in the barn for most of the time restricts grazing time, which may result in lower feed intake and milk yield [4,5] and requires the maintenance or renovation of often old infrastructures [3].

Previous studies conducted in the lowlands observed that restricting grazing time caused a decrease in milk yield associated with an increase in milk protein content, resulting in unchanged milk protein output [6]. This outcome may alter milk coagulation properties and, therefore, cheese quality. In grazing systems with restricted access to pasture, cows adapted their behavior by increasing grazing time at the expense of idling time and, more often, rumination time [7,8]. Indeed, cows can modify some behavioral traits, such as intake rate and efficiency of grazing activity, in response to short- and long-term constraints in order to control their nutrient supply [9]. In addition, a longer fasting period between grazing sessions has been reported to affect ruminal status, in particular leading to faster pH decline and increased volatile fatty acids (FA) and ammonia concentrations at the beginning of the next grazing session [10]. These changes may alter ruminal nutrient degradation and, consequently, energy supply and body fat mobilization. The sources of FA (*de novo* synthesized versus mobilized) may also influence milk’s FA composition [11]. Moreover, the exercise of cows walking back and forth to the barn, exacerbated by the effects of altitude, may affect energy balance and, ultimately, milk quality [12]. In particular, somatic cell counts were found to be elevated by walking [13], which negatively affected cheese-making properties [14].

To our knowledge, no study has comprehensively investigated the effect of different upland dairy farm management systems on the feeding behavior of dairy cows and the quality of dairy products. In 1990, Costa et al. [15] observed that milk yields were higher for cows housed part-time in a barn above 2100 m during the summer grazing season, with high day-to-day variability and no differences in herbage intake. However, that study focused more on grazing systems’ effects on the grassland (management, productivity, and fertilization) and did not deal with the consequences of different feeding behaviors on milk quality. Recent studies conducted on upland pastures during the summer grazing season investigated the effects of different concentrate amounts and divergent pasture nutritional quality on feeding behavior and milk FA composition [16,17] but did not investigate the effects of different upland farm management systems.

The present study compares the effects of part-time and full-time grazing systems on the feeding behavior of dairy cows and the consequences to milk yield, composition, and coagulation properties. One hypothesis tested was that cows with restricted rather than unrestricted access to grazing had different feeding and ruminating behaviors, lower milk and milk protein yields, and impaired milk coagulation properties due to energy deficiency. It was further hypothesized that part-time, compared to full-time, grazing on upland pastures modified the milk’s FA profile due to the different sources of substrates for milk fat synthesis. These changes may have important consequences for the sensory profiles of cheese [18].

## 2. Materials and Methods

### 2.1. Experimental Design

The experiment was conducted in the Aosta Valley (north-western Italy) on two upland farms managed according to two different grazing systems during the summer season. The experiment lasted for 19 weeks, from the beginning of April until the middle of August 2015. The farms were chosen based on their similar farm management systems during winter, calving periods, altitudes of the three respective upland grazing sites, genetic background of the cows, and because they produced the same type of cheese (Fontina PDO [19]). Both farms sheltered their cows for the entire winter in traditional tie-stall barns at 600 m above sea level (a.s.l.), where they were fed hay ad libitum and up to 5 kg of concentrate per cow per day according to their milk yield. No mineral fertilizer and no irrigation were used on the upland pastures which were fertilized only by the grazing animals’ excreta. During the entire summer grazing period, both farms offered 1 kg of a commercial concentrate (Mangime Settebello Ma. Co. Pa., Mareina & Cie, Bosconero, Italy) containing 160 g crude protein (CP), 220 g neutral detergent fiber (NDF), 30 g ether extract (EE), and 72 g total ash (TA) per kg per cow per milking on the upland grazing sites following Fontina PDO product specifications [19]. One farm (Val de Rhême, Rhêmes-Notre-Dame) was managed with a full-time grazing system, where the cows were kept exclusively on pasture and milked there (at 5:00 a.m. and 16:00 p.m.) with a mobile milking parlor. A strip grazing system was applied, with new swards allocated once daily. Pastures were limited by temporary fences, and the stocking density was 1.0 cow/ha. Water was available in troughs and creeks. The second farm (Vallone Vertosan, Avise) was managed with a part-time grazing system, with cows only grazing for 6 h per day, 4 h after the morning milking (5:00 a.m.), and 2 h after the evening milking (16:00 p.m.). This system also had a stocking density of 1.0 cow/ha, and pastures were limited by temporary fences. When not on pasture, cows were tethered in a tie-stall barn and had no access to forage. Water was available in troughs near the entrance of the barn and in creeks on the pastures. Cows were milked indoors at their stalls with portable milking devices. This part-time pasture management system is traditionally practiced on upland pastures in the Aosta Valley, and it is characteristic of, though not prescribed for, the production of Fontina PDO cheese [3]. Eight multiparous Valdostana Red Pied cows an average of 117 ± 39 (mean ± SD) days in milk (DIM) were selected from each of two herds to form two balanced groups for DIM and milk yield. At the beginning of the upland grazing time, the 16 cows had an average milk yield of 15.7 ± 2.1 kg/day, while their average fat and protein contents were 3.83 ± 0.56% and 3.40 ± 0.17%, respectively. At the beginning of the experiment, the potential ingestion capacity of the cows was calculated according to Institut National de la Recherche Agronomique (INRA) equations [20] and did not differ between the two groups (14.4 ± 1.1 kg DM/day). Three sampling periods, or grazing events (GEs), were conducted at the beginning of June (grazing event 1, GE1), middle of July (GE2), and beginning of August (GE3) 2015; cows were kept successively at three different grazing sites located at 1500, 1800, and 2000 m a.s.l., respectively. Initial sampling was performed in the barn (at 600 m a.s.l.) at the beginning of April to collect the milk yield and composition data that were included as covariates in the statistical model. Each GE was divided into two sub-sampling sessions, which each lasted for two consecutive days. Because the cows were already grazing before each GE, the first sub-sampling session took place one week after the cows arrived on the upland grazing site, and the second took place one week later. The mean seasonal rainfall in the region for the 2015 spring and summer seasons were 200 mm and 300 mm, respectively [21]. All experimental procedures complied with EU Directive 2010/63/EU and were approved by the veterinary authorities of the Regione Autonoma della Valle d’Aosta.

### 2.2. Herbage Sampling and Analyses

Representative herbage samples were collected twice during each sub-sampling session and subjected to proximate analysis. The sample strips consisted of four replicates 10 cm wide and 5 m long and were used for the measurement of biomass availability. Dry matter (DM) content was determined by oven-drying (60 °C, 72 h). The nutrient analysis included TA, CP, EE, NDF, acid detergent fiber (ADF), and acid detergent lignin (ADL), which were analyzed by near-infrared spectroscopy (NIRSystem 5000, Foss, Hillerød, Denmark) in oven-dried samples in a certified laboratory [22]. The spectrophotometer was calibrated with reference samples, which were analyzed for CP, EE, and TA according to the methods recommended by the Association of Official Analytical Chemists [23]. Contents of digestible organic matter (DOM), net energy for lactation (NE_L_), and digestible protein at the level of the duodenum according to supply with fermentable energy and rumen undegradable protein (PDIE) and according to supply with rumen degradable and undegradable protein (PDIN) were estimated according to Agroscope [24] from these nutritional values. The botanical composition of the non-grazed swards was surveyed using the point-quadrat method [25] for five plots per sward. The specific contributions of single plant species (number of individuals of each species to total number of individuals surveyed) as well as the specific contributions of grasses (*Poaceae*), legumes (*Fabaceae*), and non-legume forbs (e.g., *Apiaceae*, *Asteraceae*, etc.) were also calculated.

### 2.3. Feeding Behavior

The feeding behavior of cows on pasture was investigated during each sub-sampling session using chewing sensors and corresponding data analysis software (MSR Electronics, Henggart, Switzerland), as described by Braun et al. [26]. Animals were equipped with sensors for 24 h per sub-sampling session. Rumination bouts were characterized by uniform pressure fluctuations in the chewing sensor regularly followed by short intervals (up to 5 s) without pressure fluctuations (no jaw movements) [26]. Pressure fluctuation is highly variable during ingestion bouts, and the resulting signal waveform is irregular [26]. The variables assessed on the graphic outputs of the software were number and duration of ingestion and rumination bouts, as well as total ingestion, rumination, and idling (rest to 100%) time per day. The durations of the ingestion, rumination, and idling bouts were defined as the length of the segments of the signal waves with an uninterrupted typical signal pattern of the respective behavior. Ingestion and rumination times were calculated as the sum of all ingestion and rumination bouts, respectively, recorded during 24 h.

### 2.4. Milk Sampling and Analyses

The milking systems on both farms were equipped with the same Lactocorder^®^ (WMB AG, Balgach, Switzerland). This device provided data on individual milk yield during the sub-sampling sessions and took milk samples representative of each whole milking at each milking. Morning and evening samples of each day were pooled. Milk fat, protein, lactose, casein, urea, titratable acidity (°SH), and pH were analyzed by mid-infrared spectroscopy (MilkoScan FT6000, Foss Electric A/S, Hillerød, Denmark). The somatic cell count was determined by a fluorimetric method (Fossomatic 5000, Foss Electric, Hillerød, Denmark). The remaining milk was frozen and stored at −20 °C until being analyzed for FA, according to Koczura et al. [27]. In order to determine rennet coagulation properties, two replicates of 10 ml of fresh milk were added to 200 µL of a freshly prepared 2% *v*/*v* rennet solution (Caglio Clerici 1:10,000 with 80% chymosin and 20% pepsin, Como, Italy), following the rennet concentration used for the production of Fontina PDO cheese. Samples were incubated at 35 °C for 45 min in a Formagraph (Foss Electric A/S, Hillerød, Denmark). The measured variables were rennet coagulation time, curd firming time to reach a value of 20 mm (k_20_), and curd firmness after a strengthening time equal to the rennet coagulation time (A_R_).

### 2.5. Statistical Analyses

For statistical analysis, data were first tested for normal distribution with the Shapiro–Wilk test and then analyzed using the MIXED procedure of SAS (version 9.1 Inst. Inc., Cary, NC, USA). The model included the pasture management system (full-time or part-time grazing), grazing event (GE1, GE2, or GE3), and their interactions as fixed effects. Individual cow data from the barn were used as covariates, where available. GEs were considered repeated factors, with the cows as subjects. Multiple comparisons among means were adjusted with Tukey’s method, and effects were considered significant at *p* < 0.05 and a trend at 0.05 ≤ *p* < 0.10.

## 3. Results

### 3.1. Pasture Nutritive Value and Botanical Composition

Herbage biomass, its DM content, and nutritional value are displayed in Table 1. The CP content of the herbage was marginally higher in the full-time grazing system compared to the part-time one (138 vs. 127 g/kg DM) and decreased markedly from GE1 to GE3 in both systems. The NDF content of the herbage was similar in both systems, while the ADF and ADL contents were higher in the part-time grazing system than the full-time grazing system, except during GE3. Accordingly, the contents of NE_L_ and absorbable protein at the duodenum (both PDIE and PDIN) of the herbage were lower for part-time grazing than full-time grazing. The content of DOM was higher in the herbage of the full-time grazing system than in the part-time one during all GEs. The specific contribution of grasses, legumes, and non-legume forbs, as well as of the ten most abundant plant species in the pastures at each upland grazing site, are reported in Table 2. The full-time grazing system was characterized by a numerically lower proportion of grasses during GE2 and GE3 than the part-time grazing system.

### 3.2. Feeding Behavior

**Figure 1:**
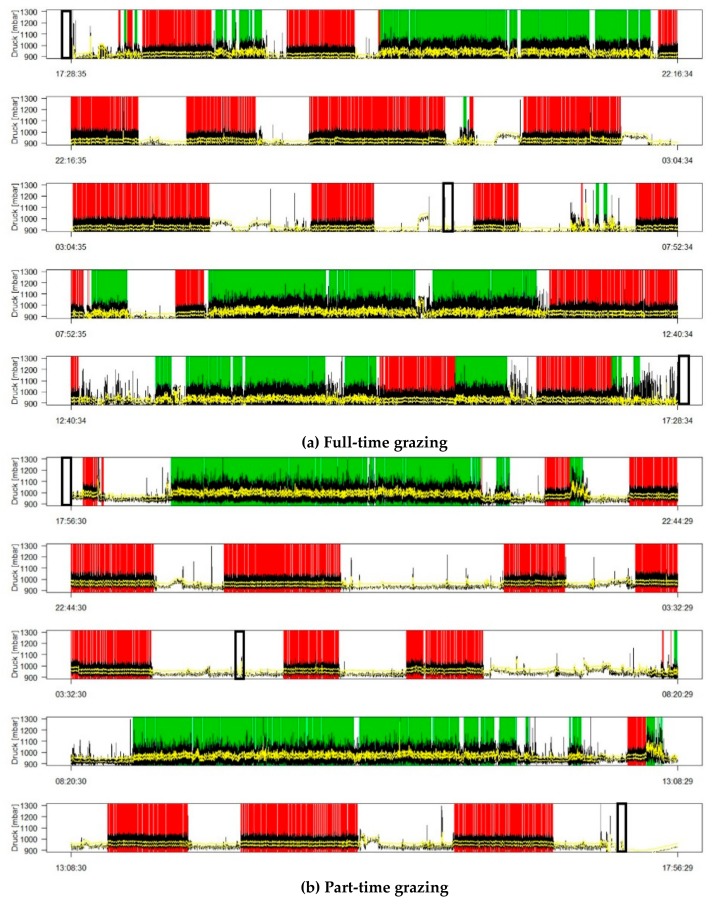
Examples of individual feeding behavior during 24 h for (**a**) full-time grazing and (**b**) part-time grazing with ingestion (green), rumination (red), and idling (white) bouts. Vertical black-lined boxes represent milking events.

Figure 1 presents examples of the feeding behavior of two cows, one from each management system, during one selected sub-sampling session (first sub-period in GE 3). Overall least square mean values per system per GE are given in Table 3.

The cows exposed to the two systems exhibited markedly different feeding behaviors (Figure 1). Part-time grazing reduced the number of ingestion bouts per day by half compared to full-time grazing (Table 3). On average, the ingestion time of part-time grazing cows was 33% shorter than full-time grazing cows. This difference was especially notable in GE1 and GE2, with part-time grazing cows having 41% and 36% shorter ingestion times, respectively, than the full-time grazing cows. Furthermore, the ingestion bouts of the part-time grazing cows lasted longer on average (+27%) than those of full-time grazing cows. Part-time grazing cows ingested during 93% of their time on pasture (337 of 360 min), while full-time grazing cows spent 35% of their time ingesting. The number of rumination bouts per day, the duration of the rumination bouts, and the total rumination time did not differ between the two systems. Rumination times increased from GE1 to GE3 by 33% for part-time grazing and 22% for full-time grazing; thus, the duration of the rumination bouts increased but not their number. Idling time and rumination bouts were longer (+34% and +72%, respectively) in part-time compared to full-time grazing. Idling time decreased from GE1 to GE3 in both systems.

### 3.3. Milk Yield, Composition, and Coagulation Properties

The average milk yield did not differ between the two systems but declined throughout the grazing season, as expected (Table 4). Milk fat content remained unchanged over the three GEs in the part-time grazing system but increased in GE3 for full-time grazing. The milk of the cows in the part-time grazing system had lower milk protein and casein contents (−2.0 g/100 g milk and −1.6 g/100 g, respectively) than the full-time grazing cows. In both systems, the lactose content decreased in GE3. During GE1, the milk’s urea content was higher by 5.9 mg/dL for part-time compared to full-time grazing, but these differences leveled out later in GE2 and GE3. During GE3, the milk’s somatic cell count and pH were lower for part-time compared to full-time grazing. Overall, rennet coagulation time, curd strengthening time (k_20_), and curd firmness (A_R_) were not affected by the system. However, A_R_ varied between GEs, and there was a trend indicating an interaction between the system and the GE.

### 3.4. Milk FA Composition

Table 5 and Table 6 report selected individual milk FA and the groups and ratios of milk FA; all other measured FA are listed in Supplementary Materials Table S1. The proportions of medium-chain saturated FA C10:0, C12:0, C14:0, and C15:0 were lower in part-time than full-time grazing by 0.39, 0.71, 1.34, and 0.44 g/100 g total FA, respectively, especially in GE1 (Table 5). Proportions of C18:1 *t*11 and C18:1 *cis* isomers, including the main oleic acid isomer (C18:1 *c*9), were higher in part-time than full-time grazing, especially in GE1 and GE2. Consequently, the C18:1 *c*9-to-C16:0 ratio was higher in part-time than full-time grazing during these GEs. The proportions of the conjugated linoleic acids (CLAs) *c*9, *c*11-CLA C18:2 and C18:3 were also lower in part-time than full-time grazing. The proportions of total saturated fatty acid (SFA) and polyunsaturated fatty acid (PUFA) were lower for part-time grazing than full-time grazing, whereas the opposite was true for the proportion of monounsaturated fatty acid (MUFA) (especially in GE1 and GE2) (Table 6). The proportion of the sum of C10 to C15 FA remained constant for part-time grazing but decreased over the three GEs for full-time grazing, where it was permanently higher than for part-time grazing. The proportion of total CLA isomers between the two systems only differed in GE3, where it was 0.58 g/kg higher in the milk fat of part-time compared to full-time grazing cows. The proportion of total n-3 FA differed between the two systems in GE2, where it increased by 20% between GE2 and GE3 for part-time grazing and by 25% between GE1 and GE2 for full-time grazing. Likewise, the proportion of total n-6 FA increased in both systems from GE1 to GE2, then remained constant in GE3. However, it never differed between the two systems. Consequently, the n-6-to-n-3 ratio was higher for part-time compared to full-time grazing during GE1 and GE2. In GE1, the proportions of odd-chain and branched-chain FA were lower in part-time compared to full-time grazing. These system differences disappeared throughout the GEs, as the proportion of odd-chain and branched-chain FA, especially of C15:0, increased from GE1 to GE3 in the part-time grazing system.

## 4. Discussion

To our knowledge, this is the first on-farm investigation of upland pastures comparing the effects of two pasture management system alternatives on the feeding behavior and milk quality of dairy cows. The upland pasture management system clearly affected cows’ feeding behavior. The longer duration of the ingestion bouts of cows on part-time grazing compared to full-time grazing is consistent with the greater eating motivation these cows were forced to exhibit in response to their limited daily grazing time [28,29]. In fact, dairy cows with time-restricted access to pasture have been reported to develop compensatory foraging strategies, such as increasing the proportion of time allocated to grazing or their intake rate or both, in order to maintain a daily target herbage intake; this was accomplished either by enlarging bite mass or increasing bite rate [28]. The longer daily idling time and number of idling bouts found in cows exposed to part-time rather than full-time grazing were consistent with their staying in the barn without access to forage. During their stay in the barn, the cows spent part of their time ruminating, but, overall, they did not ruminate longer than the cows with full-time access to pasture. The differences in ingestion behavior between systems diminished when the cows were moved to pastures of increasing altitude. This finding may have resulted from the lower amount of feed needed to cover energy and nutrient requirements due to the advancement of lactation. Concerning ingesting and ruminating behavior, the influence of herbage characteristics was relatively minor, even though the phenological stage could have a certain amount of influence on the efforts of chewing and ruminating.

Although herbage characteristics differed between the part-time and full-time grazing systems and among the GEs, these can only partly explain the differences found in milk composition and FA profiles in the concomitant GEs for both systems. In fact, rumen fermentation appeared to decrease along with the extended daily fasting periods inherent in part-time grazing, with the supply of substrates, mainly acetate, for the de novo synthesis of FA to the mammary gland being reduced. Indeed, the content of de novo synthesized FA such as C10:0, C12:0, and C14:0 was lower in the milk of part-time compared to full-time grazing cows. Nevertheless, milk yield and total milk fat content remained unaffected by the grazing system. Therefore, it can be hypothesized that the cows in the part-time grazing system mobilized body fat reserves to counterbalance the lack of energy-supplying substrates during the fasting periods. Thus, the cows were able to maintain their performance [30] and cover the energy expenditures caused by walking back and forth between the barn and the pastures [12,13]. This assumption is further supported by the higher proportion of C18:1 *c*9 found in the milk fat of the part-time compared to the full-time grazing cows because this FA is preferentially mobilized from body fat reserves [31]. The higher C18:1 *c*9-to-C16:0 ratio found in part-time compared to full-time grazing cows lowers the fat melting point, which leads to softer and creamier cheeses [32,33].

The differences in botanical composition, phenological stage, and nutrient contents of the herbage may have also affected milk composition [34]. In particular, the higher lignification and lower DOM of the herbage at the site of the part-time grazing system in GE1 and GE2, and the lower DOM, indicates that the herbage was in a more advanced phenological stage at the time of grazing than that of the full-time grazing system [35]. Moreover, in GE2 and GE3, the swards in the part-time grazing system grasses were more abundant and legumes less abundant compared to those in the full-time grazing system. Consequently, the lower milk protein and casein contents found in the part-time grazing cows are consistent with the lower NE_L_ content (lower NDF and ADF, especially in GE3) and metabolizable protein at the level of the duodenum (PDIE and PDIN) of the herbage available in the part-time grazing system. Unexpectedly, the milk coagulation properties did not differ along with the differences in milk protein and casein contents. This finding may be due to the concomitantly unaffected somatic cell count and pH, as well as the high individual variability, all of which may have masked the influence of protein and casein contents on the milk’s coagulation properties. Furthermore, the urea content in the milk of part-time grazing cows was higher during GE1, probably due to the lower supply of energy needed by rumen microbes to utilize ammonia instead of disposing of it via urea in urine and milk.

The advanced phenological stage of the herbage at the time of grazing in the part-time compared to full-time grazing system is also reflected in the higher prevalence of odd- and branched-chain FA [36,37], which resulted from the higher activity of cellulolytic bacteria in the rumen [38]. The lower proportions of MUFA such as C18:1 cis isomers C18 *t*11, intermediates of the rumen biohydrogenation of PUFA, in the full-time grazing system could be related to the different botanical compositions of the pastures in the two systems. The prevalence of dicotyledonous species like *Alchemilla gr. vulgaris*, *Polygonum bistorta*, and *Achillea millefolium*, which have relatively high contents of plant secondary compounds [39,40,41], present in the pasture grass during GE2 and GE3 in the full-time grazing system probably had an inhibitory effect on the ruminal biohydrogenation of dietary PUFA [42,43]. This effect resulted in a higher proportion of α-linolenic acid (C18:3-n3, ALA) in the milk of the cows in the full-time grazing system. Moreover, the proportion of CLA (and its most important isomer, C18:2 *c*9, *t*11) was lower in full-time than part-time grazing. However, this result may be due to the lower activity of the enzyme Δ9-desaturase in the mammary gland. However, the latter could not be confirmed by a difference in the C14:1 *c*9-to-C14:0 ratio, which is an index of desaturase activity [44]. The fact that the Δ9-desaturase activity did not differ between systems further supports the assumption that the higher proportion of C18:1 *c*9 originated from the increased mobilization of body fat reserves in the part-time grazing system.

## 5. Conclusions

This study quantified the effects of part-time grazing on upland pasture on feeding behavior and its direct consequences on milk’s FA composition. The long fasting periods caused by restricting pasture access to 6 h per day affected the rumen and energy metabolism of the cows, which involved the mobilization of body fat reserves. These findings indicate that the health and well-being of cows exposed to such a short daily grazing time may be adversely affected. However, the decision to establish or maintain one upland pasture grazing system or another also involves other boundary conditions and considerations, such as organization and availability of labor as well as the investment needed to purchase mobile milking parlors or maintain or renovate often old infrastructures. Ultimately, traditional part-time grazing systems rely on a compromise between milk and cheese quality, pasture topography, and animal and worker welfare.

## Figures and Tables

**Table 1:** Least square means of herbage biomass and nutrient composition in the two pasture management systems and the three GEs.

Item	System (S)		Full-Time Grazing (FT)			Part-Time Grazing (PT)			SEM	*p*-Values		
	FT	PT	GE1	GE2	GE3	GE1	GE2	GE3		S	GE	S × GE
Biomass (kg dry matter/m^2^)	2.18	2.74	2.41	2.06	2.07	2.43	3.27	2.52	0.40	0.059	0.531	0.220
Dry matter (DM), g/kg	251	301	162 ^b^	232 ^b^	357 ^a^	170 ^b^	354 ^a^	377 ^a^	25.9	0.014	<0.001	0.029
Nutritional value per kg DM												
Crude protein, g	138	127	155	137	121	138	123	120	7.5	0.046	0.003	0.470
Ether extract, g	28.3	28.0	26.5	28.0	30.6	24.9	29.1	30.1	1.39	0.099	0.005	0.180
Neutral detergent fiber, g	459	464	380	461	534	442	471	480	22.8	0.057	0.003	0.621
Acid detergent fiber, g	331	352	303	341	350	345	361	350	11.4	0.015	0.018	0.137
Acid detergent lignin, g	60.3	68.1	59.5	62.4	59.1	67.1	73.7	63.6	2.55	<0.001	0.013	0.310
Total ash, g	80.9	87.4	94.8 ^ab^	85.3 ^bc^	82.1 ^c^	90.3 ^ab^	81.4 ^bc^	71.1 ^bc^	4.61	0.716	0.001	0.031
DOM, g	672	632	713	660	643	641	621	635	16.7	0.008	0.057	0.196
NE_L_, MJ	5.88	5.68	6.03	5.90	5.70	5.75	5.68	5.60	0.099	0.009	0.041	0.597
PDIE, g	95.8	91.8	100.5	96.0	91.0	94.5	91.0	90.0	2.32	0.022	0.010	0.446
PDIN, g	84.2	91.6	103.3	91.0	80.4	91.8	81.4	79.3	5.06	0.048	0.003	0.471

DOM: digestible organic matter; NE_L_: net energy for lactation; PDIE: absorbable protein at the duodenum according to supply with fermentable energy and rumen undegradable protein; PDIN: absorbable protein at the duodenum according to supply with rumen degradable and undegradable protein; SEM: standard error of the mean. ^a–c^ Different superscripts within the same row indicate differences between grazing systems and GEs (*p* < 0.05).

**Table 2:** Botanical composition of the swards in the two pasture management systems (specific contributions ^1^) in each of the three GEs (bold).

Full-Time Grazing	%	Part-Time Grazing	%
**Grazing event 1**		**Grazing event 1**	
Grasses	34.9	Grasses	21.4
Legumes	20.3	Legumes	13.8
Non-legume forbs	44.8	Non-legume forbs	64.8
*Leontodon helveticus*	10.5	*Taraxacum officinale*	26.4
*Geranium sylvaticum*	9.9	*Anthriscus sylvestris*	16.4
*Dactylis glomerata*	9.3	*Dactylis glomerata*	7.5
*Poa pratensis*	7.0	*Trifolium repens*	6.9
*Trifolium pratense*	7.0	*Vicia cracca*	6.9
*Trisetum flavescens*	7.0	*Agropyron repens*	5.7
*Vicia cracca*	7.0	*Poa trivialis*	5.7
*Silene vulgaris*	6.4	*Achillea millefolium*	5.0
*Trifolium repens*	5.8	*Carum carvi*	4.4
*Anthriscus sylvestris*	4.7	*Alchemilla gr. vulgaris*	3.8
**Grazing event 2**		**Grazing event 2**	
Grasses	41.2	Grasses	44.5
Legumes	9.6	Legumes	2.9
Non-legume forbs	49.2	Non-legume forbs	52.6
*Alchemilla gr. vulgaris*	16.0	*Polygonum bistorta*	15.3
*Agrostis tenuis*	9.6	*Anthriscus sylvestris*	14.8
*Poa pratensis*	9.6	*Dactylis glomerata*	13.9
*Anthriscus sylvestris*	7.5	*Agrostis tenuis*	9.1
*Dactylis glomerata*	7.5	*Trisetum flavescens*	9.1
*Trifolium pratense*	7.0	*Rumex acetosa*	7.2
*Phleum pratense*	5.9	*Phleum alpinum*	6.2
*Polygonum bistorta*	5.9	*Achillea millefolium*	5.3
*Geranium sylvaticum*	4.8	*Poa pratensis*	5.3
*Veratrum album*	3.7	*Alchemilla gr. vulgaris*	3.3
**Grazing event 3**		**Grazing event 3**	
Grasses	39.4	Grasses	54.7
Legumes	11.1	Legumes	2.0
Non-legume forbs	49.5	Non-legume forbs	43.3
*Phleum alpinum*	17.1	*Trisetum flavescens*	20.1
*Alchemilla gr. vulgaris*	12.0	*Alchemilla gr. vulgaris*	13.3
*Poa trivialis*	9.7	*Festuca nigrescens*	9.1
*Plantago fuscescens*	6.9	*Poa pratensis*	7.3
*Polygonum bistorta*	6.0	*Phleum alpinum*	4.0
*Festuca nigrescens*	5.6	*Plantago fuscescens*	4.0
*Achillea millefolium*	5.1	*Ranunculus montanus*	3.6
*Crocus albiflorus*	4.2	*Ranunculus acris*	3.5
*Poa alpina*	4.2	*Poa alpina*	3.3
*Ranunculus pyrenaicus*	3.7	*Agrostis tenuis*	3.2

^1^ Only the ten most abundant species are displayed.

**Table 3:** Least square means of variables describing feeding and rumination behavior of cows in the two pasture management systems and the three GEs.

Item	System (S)		Full-Time Grazing (FT)			Part-Time Grazing (PT)			SEM	*p*-Values		
	FT	PT	GE1	GE2	GE3	GE1	GE2	GE3		S	GE	S × GE
Time, min/day												
Ingestion	505	337	493 ^ab^	543 ^a^	479 ^b^	289 ^d^	348 ^c^	374 ^c^	14.0	<0.001	0.001	0.001
Rumination	415	400	383	394	469	351	382	467	21.1	0.450	<0.001	0.678
Idling	482	669	547 ^c^	460 ^d^	438 ^d^	777 ^a^	664 ^b^	567 ^c^	24.2	<0.001	<0.001	0.009
Bouts, no./day												
Ingestion	16	8	17	16	14	9	9	7	0.9	<0.001	0.030	0.669
Rumination	13	13	13	14	13	13	12	13	0.7	0.214	0.931	0.488
Idling bouts	27	21	29	27	25	22	22	20	1.1	<0.001	0.050	0.634
Mean duration of bouts, min												
Ingestion	32.8	41.9	30.1	33.3	35.0	36.8	39.1	49.7	3.74	0.007	0.032	0.348
Rumination	31.9	31.6	30.2	29.3	36.2	26.9	31.5	36.3	1.64	0.872	<0.001	0.065
Idling	18.1	31.0	19.1 ^c^	18.0 ^c^	17.2 ^c^	35.9 ^a^	28.8 ^b^	28.2 ^b^	1.16	<0.001	<0.001	0.019

**Table 4:** Least square means of milk yield, composition, and milk coagulation properties in the two pasture management systems and the three GEs.

Item	System (S)		Full-Time Grazing (FT)			Part-Time Grazing (PT)			SEM	*p*-Values		
	FT	PT	GE1	GE2	GE3	GE1	GE2	GE3		S	GE	S × GE
Milk yield, kg/day	12.1	12.0	14.8	12.2	9.11	13.9	11.8	10.2	0.555	0.869	<0.001	0.086
Milk gross composition, g/100 g												
Fat	4.08	3.86	3.93 ^b^	3.70 ^b^	4.62 ^a^	3.81 ^ab^	3.87 ^ab^	3.88 ^ab^	0.202	0.295	0.009	0.014
Protein	3.65	3.45	3.65	3.57	3.75	3.45	3.40	3.49	0.094	0.049	0.267	0.859
Casein	2.87	2.71	2.86	2.79	2.94	2.73	2.68	2.71	0.072	0.002	0.354	0.554
Lactose	4.72	4.73	4.75	4.73	4.70	4.84	4.73	4.63	0.045	0.842	0.003	0.071
Urea	18.3	20.9	17.8 ^b^	19.7 ^b^	17.4 ^b^	23.7 ^a^	18.8 ^b^	20.2 ^b^	0.850	0.018	0.007	<0.001
Somatic cells, ×1000/mL	139	129	107 ^b^	122 ^b^	206 ^a^	133 ^b^	148 ^b^	108 ^b^	13.50	0.770	0.423	0.025
pH	6.68	6.64	6.69 ^a^	6.67 ^a^	6.68 ^a^	6.67 ^a^	6.65 ^a^	6.61 ^b^	0.013	0.038	<0.001	0.004
Acidity, °SH	6.57	6.73	7.16	6.34	6.21	7.10	6.52	6.56	0.162	0.369	<0.001	0.339
Coagulation properties												
Rennet coagulation time, min	18.2	18.4	18.4	17.7	18.4	17.1	18.2	19.9	2.06	0.941	0.336	0.384
k_20_, min	15.5	15.4	16.2	16.9	13.5	15.2	15.3	15.7	2.61	0.975	0.446	0.261
A_R_, mm	23.8	23.0	23.7 ^ab^	21.7 ^b^	26.1 ^a^	21.7 ^ab^	23.0 ^ab^	24.1 ^ab^	1.35	0.586	0.003	0.060

A_R_: curd firmness after a strengthening time equal to the rennet coagulation time; k_20_: curd firming time to reach a value of 20 mm; SEM: standard error of the mean. ^a,b^ Different superscripts within the same row indicate differences between grazing systems and GEs (*p* < 0.05).

**Table 5:** Least square means of proportions of selected ^1^ individual milk FA in the two pasture management systems and the three GEs.

FA, g/100g FA	System (S)		Full-Time Grazing (FT)			Part-Time Grazing (PT)			SEM	*p*-Values		
	FT	PT	GE1	GE2	GE3	GE1	GE2	GE3		S	GE	S × GE
C4:0	1.45	1.40	1.37	1.51	1.47	1.48	1.43	1.30	0.056	0.418	0.196	0.013
C6:0	1.45	1.30	1.46	1.52	1.36	1.35	1.33	1.23	0.053	0.037	0.005	0.561
C8:0	1.13	0.98	1.28 ^a^	1.14 ^b^	0.98 ^c^	1.02 ^bc^	1.00 ^bc^	0.93 ^c^	0.042	0.009	<0.001	0.004
C10:0	2.36	1.97	2.82 ^bc^	2.43 ^bcd^	1.83 ^d^	2.11 ^a^	2.00 ^b^	1.78 ^cd^	0.010	0.010	<0.001	<0.001
C10:1 *c*9	0.251	0.196	0.276 ^a^	0.265 ^a^	0.213 ^b^	0.187 ^b^	0.203 ^b^	0.199 ^b^	0.011	0.001	<0.001	<0.001
C12:0	3.00	2.33	3.73 ^a^	2.97 ^b^	2.30 ^c^	2.39 ^c^	2.42 ^c^	2.19 ^c^	0.101	<0.001	<0.001	<0.001
C12:1 *c*9	0.065	0.050	0.054	0.058	0.055	0.042	0.050	0.052	0.002	0.005	0.002	0.067
C14:0	10.71	9.62	11.99 ^a^	10.75 ^b^	9.40 ^c^	9.54 ^c^	9.94 ^bc^	9.37 ^c^	0.268	0.006	<0.001	<0.001
C14:1 *c*9	1.26	1.11	1.35 ^a^	1.23 ^b^	1.21 ^b^	0.98 ^c^	1.16 ^ab^	1.20 ^ab^	0.058	0.001	0.002	<0.001
C15:0	1.50	1.30	1.53 ^a^	1.50 ^ab^	1.50 ^ab^	1.09 ^c^	1.34 ^b^	1.47 ^a^	0.040	0.008	0.002	<0.001
C15:1 *c*9	0.365	0.339	0.363 ^ab^	0.340 ^b^	0.392 ^a^	0.286 ^c^	0.350 ^ab^	0.380 ^ab^	0.011	0.017	<0.001	0.001
C16:0	26.2	26.5	27.3 ^a^	27.1 ^a^	24.1 ^b^	27.2 ^a^	26.4 ^ab^	25.7 ^ab^	0.535	0.630	<0.001	0.025
C16:1 *c*9	0.549	0.522	0.572 ^a^	0.504 ^b^	0.571 ^a^	0.487 ^b^	0.496 ^b^	0.584 ^a^	0.015	0.155	<0.001	<0.001
C17:0	0.851	0.848	0.838	0.810	0.904	0.781	0.818	0.944	0.020	0.867	<0.001	0.120
C17:1 *c*9	0.283	0.323	0.287	0.253	0.308	0.326	0.288	0.354	0.012	0.012	<0.001	0.818
C18:0	11.1	11.3	9.8 ^c^	10.8 ^b^	12.6 ^a^	11.6 ^abc^	11.5 ^abc^	10.8 ^abc^	0.450	0.678	<0.001	<0.001
C18:1 *c*9	21.3	24.0	19.4 ^b^	20.5 ^b^	24.0 ^a^	24.1 ^a^	23.4 ^a^	24.5 ^a^	0.620	0.002	0.002	0.004
C18:1 *t*11	3.07	3.67	2.83 ^b^	3.12 ^ab^	3.27 ^a^	3.73 ^a^	3.58 ^a^	3.71 ^a^	0.153	0.018	0.060	0.017
C18:2 *c*9, *c*12 (LA)	1.66	1.91	1.48 ^c^	1.83 ^ab^	1.66 ^bc^	1.76 ^b^	1.94 ^a^	2.03 ^a^	0.061	0.003	<0.001	0.004
C18:2 *c*9, *t*11 (CLA)	1.43	1.76	1.51	1.50	1.30	1.60	1.80	1.88	0.126	0.023	0.726	0.209
C18:3n-3 (ALA)	1.102	0.897	0.937 ^bc^	1.237 ^a^	1.133 ^a^	0.784 ^c^	0.849 ^c^	1.054 ^ab^	0.050	0.003	<0.001	<0.001

CLA: conjugated linoleic acid; S: system; SEM: standard error of the mean. ^1^ For further FA, see Supplementary Table S1. ^a–d^ Different superscripts within the same row indicate differences between grazing systems and GEs (*p* < 0.05).

**Table 6:** Least square means of proportions of sums and ratios of milk FA in the two pasture management systems and the three GEs.

FA, g/100g FA	System (S)		Full-Time Grazing (FT)			Part-Time Grazing (PT)			SEM	*p*-Values		
	FT	PT	GE1	GE2	GE3	GE1	GE2	GE3		S	GE	S × GE
SFA	61.4	59.1	63.8 ^a^	62.2 ^ab^	58.3 ^c^	59.8 ^bc^	59.8 ^bc^	59.6 ^c^	0.70	0.009	<0.001	0.017
MUFA	32.1	34.8	30.3 ^b^	31.0 ^b^	35.1 ^a^	34.7 ^a^	34.1 ^a^	35.7 ^a^	0.61	0.002	<0.001	0.002
PUFA	6.40	6.11	5.89 ^bc^	6.78 ^a^	6.54 ^ab^	5.55 ^c^	6.10 ^abc^	6.67 ^ab^	0.203	0.234	<0.001	0.037
∑ CLA ^1^	1.52	1.84	1.58 ^bc^	1.59 ^bc^	1.39 ^c^	1.68 ^b^	1.88 ^ab^	1.97 ^a^	0.136	0.087	0.479	0.037
n-3	1.38	1.15	1.21 ^bc^	1.52 ^a^	1.42 ^a^	1.00 ^c^	1.11 ^c^	1.33 ^ab^	0.059	0.004	<0.001	0.002
n-6	2.46	2.65	2.21	2.61	2.56	2.47	2.69	2.78	0.086	0.100	<0.001	0.228
Σ C10 to C15 ^2^	20.8	17.9	25.5 ^a^	23.0 ^b^	20.0 ^c^	19.9 ^c^	20.6 ^bc^	19.4 ^c^	0.563	0.001	<0.001	<0.001
Odd-chain FA ^3^	3.22	2.99	3.26 ^ab^	3.09 ^ab^	3.30 ^a^	2.64 ^c^	2.99 ^b^	3.36 ^a^	0.065	0.011	<0.001	<0.001
Branched-chain FA ^4^	1.72	1.47	1.69 ^ab^	1.71 ^ab^	1.75 ^a^	1.30 ^c^	1.50 ^b^	1.60 ^ab^	0.054	0.003	<0.001	<0.001
Σ C18:1 *cis* ^5^	22.4	25.2	20.9 ^b^	21.4 ^b^	25.0 ^a^	25.3 ^a^	24.6 ^a^	25.7 ^a^	0.559	<0.001	<0.001	<0.001
n-6/n-3	1.83	2.31	1.89 ^bc^	1.76 ^c^	1.85 ^bc^	2.44 ^a^	2.42 ^a^	2.08 ^b^	0.053	<0.001	<0.001	<0.001
C18:1 *c*9/C16:0	0.92	0.82	0.71 ^b^	0.75 ^b^	0.99 ^a^	0.90 ^a^	0.90 ^a^	0.97 ^a^	0.033	0.012	<0.001	<0.001
C14:1 *c*9/C14:0	0.113	0.115	0.120	0.115	0.108	0.118	0.115	0.111	0.005	0.839	0.177	0.961

MUFA: monounsaturated FA; P: Period; PUFA: polyunsaturated FA; S: system; SEM: standard error of the mean; SFA: saturated FA. ^1^ Includes C18:2 *c*9, *t*11, C18:2 *c*9, *c*11 and C18:2 *t*9, *t*11. ^2^ Includes C10:0, C10:1 *c*9, C12:0, *iso*-C12:0, C12:1 *c*9, C13:0, *iso*-C13:0, C14:0, C14:1 *c*9, *iso*-C14:0, *anteiso*-C14:0, C15:0, *iso*-C15:0, C15:1 *c*9. ^3^ Includes C13:0, C15:0, C17:0 and C19:0 (see also Supplementary Table S1). ^4^ Includes *iso*-C12, *iso*-C13, *iso*-C14, *anteiso*-C14, *iso*-C15, *iso*-C16:1, *anteiso*-C16, *iso*-C17, and *anteiso*-C17:1 (see also Supplementary Table S1). ^5^ Includes C18:1 *c*9, C18:1 *c*10, C18:1 *c*12 and C18:1 *c*13 (see also Supplementary Table S1). ^a–c^ Different superscripts within the same row indicate differences between grazing systems and GEs (*p* < 0.05).

## Supplementary Materials

The following are available online at https://www.mdpi.com/2076-2615/9/11/908/s1, Table S1: Least square means of the proportions of the FA in milk fat not listed in Table 5 in the two pasture management systems and the three GEs.
