# Supplementary material for: Grazing on Upland Pastures Part-Time Instead of Full-Time Affects the Feeding Behavior of Dairy Cows and Has Consequences on Milk Fatty Acid Profiles
Source: Animals (Basel). 2019 Nov 1;9(11):908. doi: 10.3390/ani9110908 (PMC6912772; doi:10.3390/ani9110908)
Supplement: Supplementary file 1 [file animals-09-00908-s001.pdf]

Table S1. Least square means of the proportions of the FA in milk fat not listed in Table 5 in the two pasture management systems and the three GEs.

Fatty Acids	System (S)		Full-Time Grazing (FT)			Part-Time Grazing (PT)			SEM	*p*-Values		
g/100 g FA	FT	PT	GE1	GE2	GE3	GE1	GE2	GE3		S	GE	S × GE
C13:0	0.114	0.092	0.134 ^{a}	0.104 ^{b}	0.104 ^{b}	0.077 ^{c}	0.097 ^{b}	0.101 ^{b}	0.002	<0.001	0.108	<0.001
iso-C13:0	0.196	0.183	0.181 ^{b}	0.190 ^{b}	0.217 ^{a}	0.135 ^{c}	0.198 ^{ab}	0.216 ^{ab}	0.008	0.178	<0.001	0.001
anteiso-C14:0	0.766	0.645	0.752 ^{ab}	0.739 ^{ab}	0.806 ^{a}	0.510 ^{c}	0.678 ^{b}	0.746 ^{a}	0.024	<0.001	<0.001	<0.001
iso-C15:0	0.087	0.068	0.075 ^{bc}	0.103 ^{a}	0.082 ^{bc}	0.055 ^{d}	0.068 ^{c}	0.082 ^{b}	0.004	0.004	<0.001	<0.001
iso-C16:0	0.255	0.255	0.238 ^{ab}	0.278 ^{ab}	0.248 ^{ab}	0.267 ^{b}	0.251 ^{a}	0.247 ^{b}	0.008	0.986	0.043	0.001
C18:1 *t*6/7/8 [1]	0.293	0.335	0.233 ^{c}	0.270 ^{bc}	0.375 ^{a}	0.317 ^{ab}	0.350 ^{ab}	0.340 ^{ab}	0.018	0.100	<0.001	<0.001
C18:1 *t*10	0.359	0.359	0.315 ^{d}	0.361 ^{c}	0.401 ^{ab}	0.394 ^{abc}	0.428 ^{a}	0.359 ^{bcd}	0.014	0.070	<0.001	<0.001
C18:1 *t*12	0.347	0.329	0.325 ^{a}	0.360 ^{a}	0.354 ^{a}	0.349 ^{a}	0.355 ^{a}	0.284 ^{b}	0.019	0.497	0.002	<0.001
C18:1 *c*11	0.759	0.830	0.691 ^{c}	0.732 ^{bc}	0.854 ^{a}	0.847 ^{a}	0.802 ^{ab}	0.841 ^{a}	0.024	0.003	<0.001	<0.001
C18:1 *c*12	0.130	0.150	0.105 ^{b}	0.116 ^{b}	0.167 ^{a}	0.129 ^{b}	0.162 ^{a}	0.160 ^{a}	0.006	0.023	<0.001	<0.001
C18:1 *c*13	0.129	0.107	0.127	0.127	0.129	0.109	0.106	0.105	0.008	0.068	0.924	0.829
C18:1 *c*14 + *t*16	0.316	0.260	0.270 ^{c}	0.324 ^{ab}	0.355 ^{a}	0.251 ^{c}	0.253 ^{c}	0.276 ^{bc}	0.011	0.001	<0.001	0.004
C18:2 *t*9, *t*12	0.480	0.442	0.433 ^{b}	0.452 ^{ab}	0.555 ^{a}	0.453 ^{ab}	0.443 ^{ab}	0.432 ^{b}	0.028	0.278	0.063	0.009
C18:2 *c*9, *c*11	0.064	0.039	0.052 ^{bc}	0.078 ^{a}	0.062 ^{b}	0.034 ^{c}	0.043 ^{bc}	0.041 ^{bc}	0.006	0.006	<0.001	0.023
C18:2 *t*9, *t*11	0.033	0.033	0.031	0.033	0.037	0.029	0.033	0.037	0.003	0.928	0.015	0.365
C18:2 *c*9, *t*12	0.054	0.045	0.024 ^{b}	0.057 ^{a}	0.079 ^{a}	0.051 ^{ab}	0.041 ^{ab}	0.043 ^{ab}	0.009	0.460	0.010	<0.001
C18:2 *t*9, *c*12	0.597	0.494	0.491 ^{cd}	0.615 ^{ab}	0.684 ^{a}	0.428 ^{d}	0.451 ^{bd}	0.603 ^{ac}	0.035	0.040	<0.001	0.042
C18:2 *c*9, *c*15	0.169	0.153	0.155	0.165	0.186	0.144	0.154	0.161	0.008	0.175	<0.001	0.116
C18:3n-6	0.057	0.049	0.053	0.057	0.062	0.048	0.045	0.053	0.004	0.032	0.058	0.509
C19:0	0.099	0.097	0.101 ^{ab}	0.082 ^{b}	0.113 ^{a}	0.087 ^{ab}	0.096 ^{ab}	0.108 ^{ab}	0.006	0.800	<0.001	0.034
C20:1 t	0.056	0.034	0.067 ^{a}	0.077 ^{a}	0.023 ^{b}	0.025 ^{b}	0.051 ^{a}	0.027 ^{b}	0.006	0.003	<0.001	<0.001
C20:1 c5	0.128	0.101	0.110	0.146	0.127	0.080	0.129	0.094	0.010	0.040	<0.001	0.582
C20:1 c9	0.156	0.164	0.110 ^{c}	0.135 ^{b}	0.221 ^{a}	0.119 ^{bc}	0.129 ^{bc}	0.244 ^{a}	0.008	0.422	<0.001	0.011
C20:1 c11	0.080	0.070	0.055 ^{d}	0.078 ^{bc}	0.108 ^{a}	0.068 ^{cd}	0.054 ^{d}	0.093 ^{ab}	0.005	0.085	<0.001	<0.001
C20:2n-6	0.075	0.068	0.065	0.077	0.083	0.055	0.072	0.078	0.004	0.093	<0.001	0.531
C20:3n-3	0.079	0.079	0.068	0.083	0.085	0.066	0.082	0.088	0.004	0.971	<0.001	0.471
C20:3n-6	0.048	0.040	0.046	0.049	0.048	0.031	0.045	0.042	0.004	0.070	0.062	0.267
C20:4n-3	0.064	0.054	0.065	0.067	0.060	0.052	0.054	0.056	0.003	0.016	0.707	0.237
C20:4n-6	0.071	0.072	0.059	0.083	0.071	0.065	0.073	0.077	0.005	0.867	0.006	0.165
C20:5n-3	0.099	0.085	0.089 ^{c}	0.109 ^{a}	0.099 ^{b}	0.070 ^{d}	0.091 ^{bc}	0.094 ^{bc}	0.003	<0.001	<0.001	0.003
C22:0	0.102	0.108	0.078 ^{d}	0.103 ^{c}	0.124 ^{b}	0.070 ^{d}	0.106 ^{bc}	0.144 ^{a}	0.004	0.323	<0.001	0.001

C22:1 c9	0.162	0.169	0.179	0.166	0.141	0.155	0.171	0.180	0.012	0.596	0.705	0.018
C22:4n-6	0.039	0.035	0.036	0.037	0.044	0.024	0.038	0.044	0.003	0.360	<0.001	0.028
C22:5n-3	0.009	0.008	0.010	0.009	0.009	0.007	0.008	0.008	0.002	0.464	0.973	0.603
C22:5n-6	0.041	0.028	0.049^{a}	0.033^{c}	0.036^{b}	0.030^{cd}	0.030^{cd}	0.024^{d}	0.002	<0.001	<0.001	<0.001
C22:6n-3	0.030	0.023	0.038	0.018	0.036	0.021	0.020	0.028	0.008	0.451	0.048	0.246

SEM: Standard error of the mean. $^{a–d}$ Within the same variable, means without a common superscript letter are significantly different according to Tukey's test ($p < 0.05$).
[1] C18:1 *t*6, C18:1 *t*7, and C18:1*t*8 could not be differentiated with the chromatographic procedure applied.
